# Development of Neovasculature in Axially Vascularized Calcium Phosphate Cement Scaffolds

**DOI:** 10.3390/jfb14020105

**Published:** 2023-02-14

**Authors:** Yassine Ouhaddi, Baptiste Charbonnier, Juliette Porge, Yu-Ling Zhang, Isadora Garcia, Uwe Gbureck, Liam Grover, Mirko Gilardino, Edward Harvey, Nicholas Makhoul, Jake Barralet

**Affiliations:** 1 Division of Orthopaedics, Department of Surgery, Faculty of Medicine and Health Sciences, Montreal General Hospital, Montreal, QC H3G 1A4, Canada; 2 Faculty of Dentistry, McGill University, 2001 McGill College Avenue, Montreal, QC H3A 1G1, Canada; 3 Division of Operative Dentistry, Department of General Dentistry, University of Maryland School of Dentistry, Baltimore, MD 21201, USA; 4 Department of Functional Materials in Medicine and Dentistry, University of Würzburg, D-97070 Würzburg, Germany; 5 School of Chemical Engineering, University of Birmingham, Birmingham B15 2TT, UK

**Keywords:** angiogenesis, axial vascularization, bioceramic, bioinorganic, calcium phosphate, NLRP3, inflammation

## Abstract

Augmenting the vascular supply to generate new tissues, a crucial aspect in regenerative medicine, has been challenging. Recently, our group showed that calcium phosphate can induce the formation of a functional neo-angiosome without the need for microsurgical arterial anastomosis. This was a preclinical proof of concept for biomaterial-induced luminal sprouting of large-diameter vessels. In this study, we investigated if sprouting was a general response to surgical injury or placement of an inorganic construct around the vessel. Cylindrical biocement scaffolds of differing chemistries were placed around the femoral vein. A contrast agent was used to visualize vessel ingrowth into the scaffolds. Cell populations in the scaffold were mapped using immunohistochemistry. Calcium phosphate scaffolds induced 2.7–3 times greater volume of blood vessels than calcium sulphate or magnesium phosphate scaffolds. Macrophage and vSMC populations were identified that changed spatially and temporally within the scaffold during implantation. NLRP3 inflammasome activation peaked at weeks 2 and 4 and then declined; however, IL-1β expression was sustained over the course of the experiment. IL-8, a promoter of angiogenesis, was also detected, and together, these responses suggest a role of sterile inflammation. Unexpectedly, the effect was distinct from an injury response as a result of surgical placement and also was not simply a foreign body reaction as a result of placing a rigid bioceramic next to a vein, since, while the materials tested had similar microstructures, only the calcium phosphates tested elicited an angiogenic response. This finding then reveals a potential path towards a new strategy for creating better pro-regenerative biomaterials.

## 1. Introduction

Significant progress has been made in developing several substitutes for non-vascularized bone autografts [1,2]. However, the primary effective repair for large segmental bone defects (SBD) is microsurgically transplanted vascularized bone autograft (VBA). Non-vascularized autograft repair of SBD is associated with high failure rates (e.g., 75% at >12 cm, compared with 5% for VBA) [3,4]. Vascularization reduces the infection rate [5,6]. VBA can provide up to 25 cm of straight, cortical bone with a vascular pedicle without sacrificing an otherwise normal anatomical area. While revolutionary in 1975 [7], clear disadvantages of VBA remain: (i) additional trauma and associated morbidity [8], (ii) longer hospitalization [9], and (iii) anatomical mismatch between donor and recipient site hindering the restoration of normal function.

The vascular supply, central to most tissue-regenerative strategies, has so far been resistant to attempts at augmentation. Capillaries proliferate in and around implanted tissues and materials. Still, their capacity to nourish tissue volumes is limited, and they either remodel and mature or regress if they are not serving a tissue-sustaining function [10,11]. Large-diameter vessels have not been shown to spontaneously regenerate or sprout to form new angiosomes to sustain new engineered tissues. Revascularization is still only performed surgically, mainly using vein grafts anastomosed to an artery as conduits. Vein tissue is preferred because it is dispensable and can be harvested without causing irreparable harm [12].

One of the most compelling demonstrations of surgically induced vascular regeneration leading to in vivo formation of a functional VBA relies on a surgical technique known as the arteriovenous loop (AVL). It is thought that when a thin-walled vein is grafted to an artery, the high arterial blood pressure causes new vessels to sprout from the transplanted vein. When combined with osteogenic agents, these new vessels can be used to create custom VBAs that have shown clinical success [13]. Although effective, in its current form, it is complex and surgically demanding.

Our group recently reported a calcium phosphate-mediated angioinduction [14], which was a preclinical proof of concept that suggested that microsurgical arterial anastomosis of a large-diameter AVL was unnecessary to induce luminal sprouting of large-diameter vessels [14,15]. We also demonstrated that using this technique to vascularize marrow aspirate improved bone formation capacity over non-vascularized constructs (66+/−6% vs. 30+/−4%) [15]. Only in the presence of marrow were osteoclasts detected in these subcutaneous implants [15], suggesting that blood vessels were remodelling the cements by another mechanism. Macrophages are among the most abundant phagocytotic cells found at areas of inflammation, and they are known to be necessary for bone regeneration and angiogenesis mediated by bioceramics [16].

While exciting and clearly hugely relevant to overcoming challenges in tissue regeneration, gaps in knowledge were highlighted in our prior studies. Was luminal sprouting simply caused by the injury caused by handling the vessel surgically, or was it a foreign body response to having a stiff microporous microcrystalline biomaterial matrix placed around the vessel? In order to address these questions, in this study, we observed markers of sterile inflammation over time and implanted a variety of materials with different chemistries but largely similar physical microstructures.

It has been observed that angiogenesis of calcified vascular plaque pathologies is an important step in the progression of plaque instability and so is a matter of considerable research [17,18]. Most research in this field considers the calcification to be simply a stage of plaque development, and this work is, to our knowledge, the first to study the angiogenic response of veinous adventitia to a variety of bioceramics.

In this study, using micro-CT and immunohistochemistry (IHC), we examined the temporal changes in and around scaffolds that accompanied luminal sprouting of veins into monetite cements. In a pilot study, we sought to determine if sprouting was a general response to surgical injury and placement of an inorganic construct around the vessel. A better understanding of material characteristics that induce the sprouting of large-diameter vessels could ultimately provide a path to better pro-regenerative materials with which to repair and reconstruct missing tissues.

## 2. Materials and Methods

### 2.1. Formulation of Experimental Scaffolds

Monetite cement was made as described previously [19]; in brief, water was added to a powder mixture of β tricalcium phosphate (β-TCP, Ca_3_(PO_4_)_2_) (Sigma–Aldrich, MO, USA, 7758874) and monocalcium phosphate monohydrate (MCPM, Ca(H_2_PO_4_)_2_·H_2_O) (ABCR, Karlsruhe, Germany, AB119788) at a molar ratio of 1.2. This cement initially formed brushite, and after drying, the cements were autoclaved to dehydrate them to create monetite.

Calcium sulphate dihydrate, CaSO_4_·2H_2_O (Gypsum), was made by mixing calcium sulphate hemihydrate CaSO_4_·0.5H_2_O (Crystacal R, British Gypsum, Loughborough, UK) with deionized water. To synthesize magnesium phosphate cements, the general method of Kingery [20] was followed. Calcined MgO (Thermo, MA, USA, AC205155000) and 20 wt.% H_3_PO_4_ (Fisher, MA, USA, 7664382) were mixed to form a rapidly setting cement.

The powder-to-liquid (P:L) ratios used are shown in Table 1. The P:L ratio was previously determined such that levels of microporosity among the four cements were within the same range (50 ± 5%).

**Figure 1:**
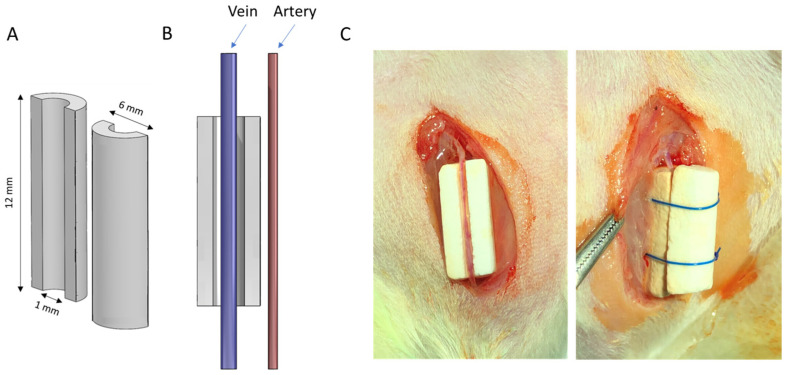
(**A**) Schematic diagram of scaffold geometry and (**B**) positioning of the vein axially within implant. (**C**) Photographs of the surgical procedure showing stages in scaffold assembly.

Cylindrical cement samples (12 ± 0.1 mm high, 6 ± 0.1 mm external diameter, and 1 mm internal diameter) were prepared by placing the cement pastes into a half-cylindrical dental silicone mould immediately after mixing. A 1 mm diameter polytetrafluoroethylene (PTFE) cylinder was positioned on the centre of the half-cylinder to create the impression of an internal channel in the cement as it set. In this way, two hemi-cylinders could be assembled around a blood vessel with 4.0 Prolene^®^ polypropylene sutures (Ethicon, NJ, USA, 8695) (Figure 1). After setting, the samples were removed from the mould, and the PTFE cylinder was carefully removed. The half-cylinders were kept at 40 °C in a dry environment under a low vacuum (10 mmHg) overnight.

### 2.2. Scaffold Characterization

The set cements were characterized by X-ray diffraction (XRD) analysis to determine the crystalline phase composition by matching with standard database patterns. Micro-computed tomography (μCT) was used to analyse cement porosity (*n* = 4). The materials were ground with a pestle and sieved for X-ray diffraction analysis. The diffractometer (Siemens D5005, Siemens, Karlsruhe, Germany) was operated at 40 kV, Cu K-α radiation, 40 mA, step size of 0.02° over a range 10°–80° 2θ, with an acquisition time of 300 s per frame.

The scaffold architecture was investigated with μCT (SkyScan 1172, SkyScan Kontich, Belgium) with a resolution of 10.9 µm at 40 kV and an intensity of 250 µA (0.5 mm aluminium filter). The strut density of the scaffolds was determined using a helium pycnometer (Accupyc 1330, Micromeritics, UK). The global porosity was calculated as the ratio of the apparent density to the bulk density.

Cement microstructures were characterized by scanning electron microscopy (SEM), and porosity was measured by mercury porosimetry. The samples were placed on metallic stubs and gold-sputter-coated. SEM (Hitachi S-4700 FE-SEM; Tokyo, Japan) was used to capture microstructural images using an accelerating voltage of 20 kV. Mercury porosimetry (Micromeritics 9420) was used to determine the pore size distribution of the cements.

### 2.3. In Vitro Evaluation of Scaffold Dissolution

Five cylindrical samples of 12 mm × 6 mm per cement type were prepared for dissolution analysis. Each sample was stored in 50 mL of phosphate-buffered saline (PBS) (Fisher, USA, 10010049) solution at 37 °C. The weight of each sample was measured every 3 days after carefully removing excess water with a damp paper towel, and the PBS was replaced after each weighing for 60 days.

### 2.4. Animal Model and Surgical Procedure

The in vivo study was carried out with male Wistar rats (400 to 600 g) at Charles River Laboratory following institutional guidelines for humane animal treatment and complied with the legislation of the McGill University Animal Care Committee, who approved this protocol (UACC, #7662). The surgical procedure was performed according to a previous study [15]. In summary, the femoral vein in both lower limbs was gently dissected from the arteriovenous vascular bundle under a microsurgical microscope (Leica TC12, Leica Biosystems GmbH, Nussloch, Germany). One-half of the scaffold samples were positioned under the vein, which was positioned within the central channel of the sample. The second half was placed on top, and both parts were kept together with 4.0 PROLENE^®^ sutures (Figure 1). Special care was taken not to pinch the vein between the two halves of the scaffold. The surgical sites were sutured closed using a 4.0 monocryl wire (Ethicon, USA, Y823). The rats were induced and maintained under isoflurane anaesthesia during the whole procedure. As per UACC SOP, carprofen was administered (0.1 mL/100 g weight) every 24 h for 3 days for analgesia, since it is an effective pain reliever for small animals, particularly for post-operative pain that can be used at relatively high doses without causing significant side effects [21,22].

After 1, 2, 4, and 6 weeks of implantation, the animals were sacrificed and perfused with contrast agent, which polymerized inside their vascular network. The heart was exposed by opening the ribcage. Heparinized PBS (150 mL) followed by 4% paraformaldehyde (150 mL) was injected into the left ventricle by gravity flow via a perfusion bag set with an 18G needle. A small incision in the right atrium allowed exsanguination. Yellow Microfil (MV-122, Flowtech, CO, USA) injection was performed in the descending aorta and stopped when the fluid reached the heart through the right atrium. The descending aorta and inferior vena cava were then ligated, and the Microfil was allowed to harden overnight in the refrigerator at 4 °C. Finally, explants were harvested and placed in 4% PFA for 12 h, rinsed with PBS, and stored in 70% ethanol.

### 2.5. Evaluation of Scaffold Dissolution and Blood Vessel Density in the Animal Explants

To evaluate the in vivo degradation of the scaffolds, the explants were analysed by μCT using the same parameters as described for the scaffold characterization. The inorganic content was quantified with CT-Analyser software. Then, the samples were placed for 3 to 5 weeks in 14 wt.% of ethylenediaminetetraacetic acid (EDTA) at pH 7.2 to gently dissolve the inorganic phases and allow better visualization of the developed vascular network filled with insoluble Microfil. The total blood vessel density within the scaffolds was determined using CT-Analyser. CT-Analyser was also used to evaluate the blood vessel density.

### 2.6. Immunohistochemical (IHC) Staining

Decalcification of explants (*n* = 3) was performed in ethylenediaminetetraacetic acid (EDTA, 14 wt.%) at pH 7.2 for several weeks at room temperature until the samples were radiolucent. Dehydration in ascending serial ethanol solutions preceded paraffin embedding, followed by sectioning into 4 µm histological slices with a microtome (SP 1600 microtome Leica Microsystems, Germany). NLRP3 (1:200 dilution Thermo Fisher, PA5-79740), IL-1 beta (1:200 dilution Novus, NB600-633), IL-8 (1:200 dilution Proteintech, 27095-1-AP), Anti-F4/80 (1:500 dilution Abcam, ab300421), CD-34 (1:200 dilution Thermo Fisher, MA5-18091), Calponin-1 (1:100 dilution Santa Cruz; sc-58707), and α-smooth muscle actin (1:2000 dilution eBioscience 14-9760-82) staining was performed and incubated with a secondary antibody using the OmniMap anti-Rb HRP (RUO) DISCOVERY, (CiteAb, Bath, UK). The colour was developed with 3,3′-diaminobenzidine (DAB) (Vector Laboratories, Newark, CA, USA). The slides were counterstained with haematoxylin. Immunohistological imaging was performed using a Leica scanner Aperio ScanScope XT.

### 2.7. Statistical Analysis

Quantitative data were analysed by comparing the significance of differences between means using the StatPage calculator (http://statpages.info/anova1sm.html, accessed on 15 December 2022). One-way ANOVA was performed followed by the Tukey post hoc test to compare the four groups of experimental scaffolds. The probablilty level of 5% was considered significant. Descriptive analysis was performed for the XRD patterns and for the images acquired.

## 3. Results

**Figure 2:**
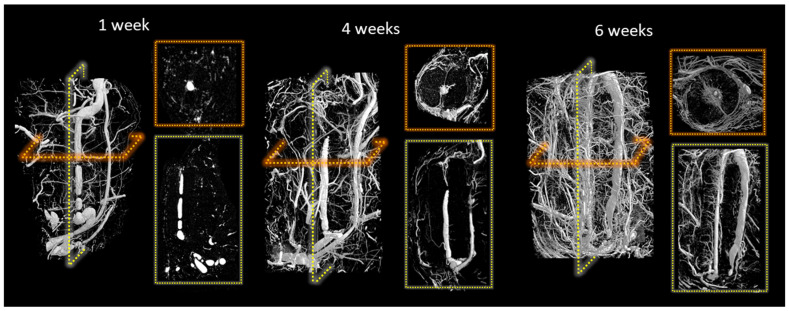
Micro-CT images of perfused blood vessels in decalcified scaffolds showing development of vasculature inside monetite scaffolds.

At the end of the study periods, the vascular system was infused with radio-opaque contrast agent. IHC staining for CD34 confirmed endothelial cell lining around these vessels (Figure S1). After harvest, the scaffolds were decalcified, allowing microCT imaging of the vasculature that had penetrated the scaffolds. Figure 2 shows the development of new vessels sprouting from the femoral vein in monetite scaffolds during the implantation. One week after implantation, a vascularized membrane had developed around the outside of the scaffold, but little vessel ingrowth was evident. By week 4, the inorganic substrate was remodelled as new blood vessels grew through the material and luminal sprouting was evident. At week 6, the most apparent change was the increase in vascular density within the scaffold, and blood vessels now appeared to be entering the scaffold from the outer periphery.

**Figure 3:**
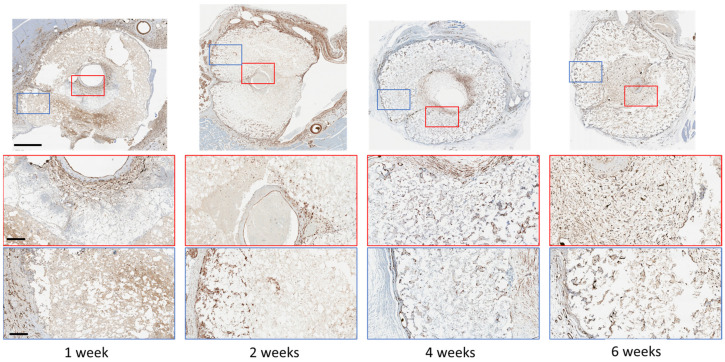
Decalcified immunohistochemistry (IHC) staining of alpha-smooth muscle cell expression at low and high magnification, outside and within the scaffold. Scale bars on low and high magnification represent 1 mm and 400 µm, respectively.

Of the several cell types found on the adventitia of veins, we sought to establish if smooth muscle cells were colonizing the microporosity inside the monetite. After 1 week, we found SMA-positive staining in the space between the central vein and the scaffold. After 2 weeks, isolated groups of SMA-expressing cells could be detected inside the scaffold, and by 4 weeks, these cells could be seen encircling small regions that appeared to be blood vessels throughout the cement thickness. The density of these cells then increased after 6 weeks, and in this sample, Microfil was present inside some of these structures (visible as black residue in Figure 3), confirming that these were indeed blood vessels.

**Figure 4:**
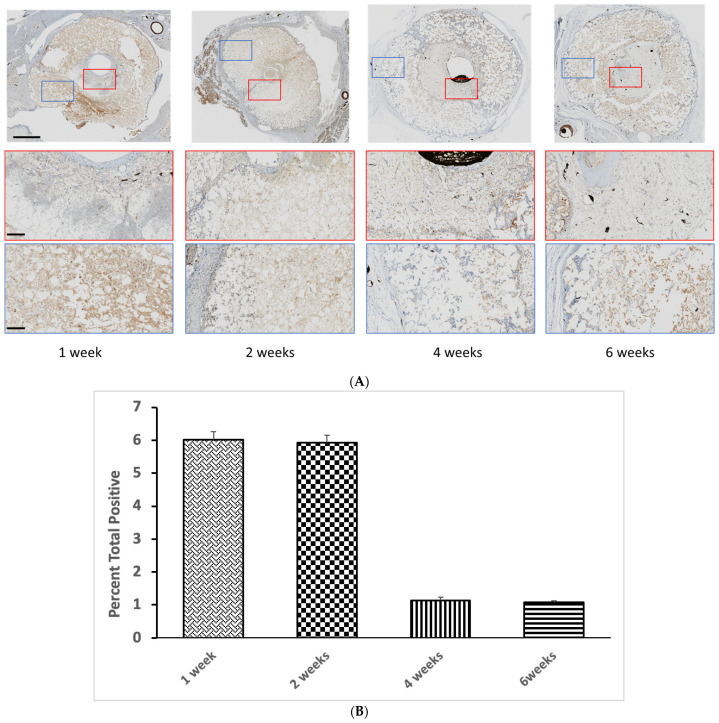
(**A**) Decalcified immunohistochemistry (IHC) staining of Calponin-1 expression at low and high magnification, outside and within the scaffold. Scale bars on low and high magnification represent 1 mm and 400 µm, respectively. (**B**) Quantification of Calponin-1 staining after different implantation times. Bars represent means ± std. dev. (*n* = 3).

As smooth muscle actin is not a specific marker for vascular smooth muscle cells (vSMC), we also examined whether Calponin-1 was expressed by cells in the scaffolds, since Calponin-1 is specifically expressed in smooth muscle cells [23]. We found a broadly similar distribution of expression as for SMA (Figure 4). Consecutive sections from the same paraffin block with a-SMA and Calponin-1 antibody indicated co-localisation of at least a population of the cells inside the scaffold (Figure S2), suggestive of vSMC. Quantification indicated a reduction in vSMC after 4 weeks, suggesting a proliferation followed by a concentration of vSMC around vessels as the blood vessels matured.

**Figure 5:**
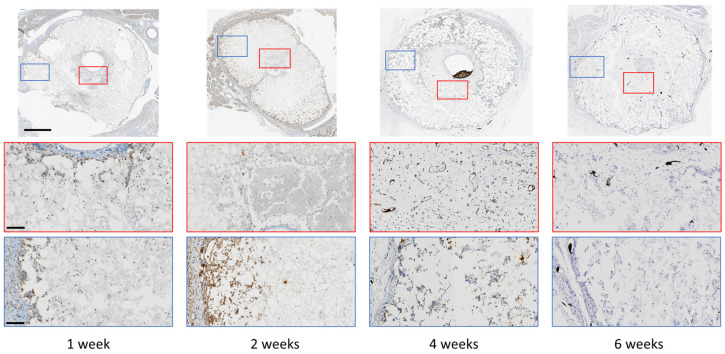
Decalcified immunohistochemistry (IHC) staining of F4/80-expressing cells at low and high magnification, outside and within the scaffold. The black material inside the vessels at 4 weeks is Microfil contrast agent. Scale bars on low and high magnification represent 1 mm and 400 µm, respectively.

Inorganic cements are essentially an interlocking mass of nano- and micro-crystals, and macrophages are amongst the cell populations known to accumulate during the initial inflammatory stage of healing. We stained decalcified sections for F4/80, a marker of macrophages. After 1 week, macrophages were observed at similar concentrations at the outer and inner surfaces. However, after 2 weeks, no macrophages were observed on the inner surface, and they could be observed mainly within the outer region of the cement. After 4 weeks, macrophages could be observed sparsely distributed through the scaffold, but by 6 weeks, no macrophages were observed in the material (Figure 5).

**Figure 6:**
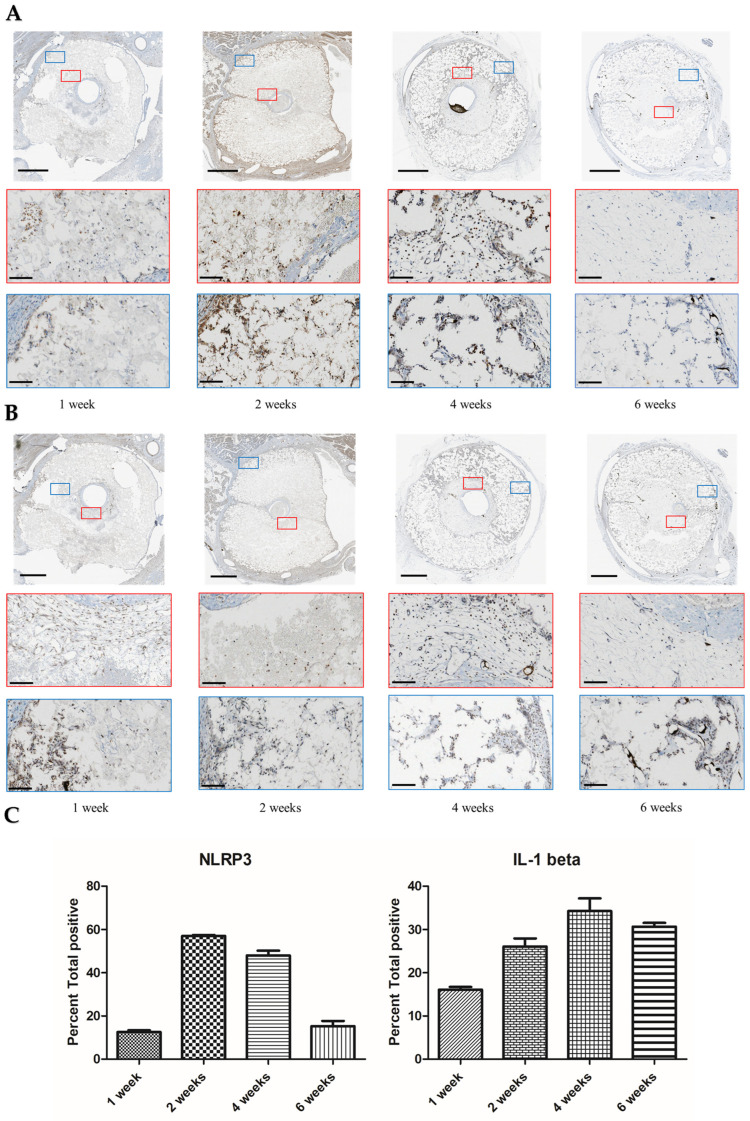
(**A**) Decalcified immunohistochemistry (IHC) staining of NLRP3 inflammasome, and (**B**) Il-1 beta expression at low and high magnification, outside and within the scaffold. Scale bars on low and high magnification represent 1 mm and 100 µm, respectively. (**C**) Quantitative analysis of NLRP3 and IL-1β staining after different implantation times. Bars represent means ± std. dev (*n* = 3).

We suspected that crystals released from the cements may have activated the NLRP3 inflammasome, which would have resulted in an expression of IL-1β. We observed an increase in expression of NLRP3 after 2 weeks that became greatly reduced by 6 weeks. Expression of Il-1β appeared from the first week at the inner and outer surfaces of the cements and persisted for at least 6 weeks (Figure 6). The IHC was quantified using image analysis of the decalcified region (Figure 6C).

**Figure 7:**
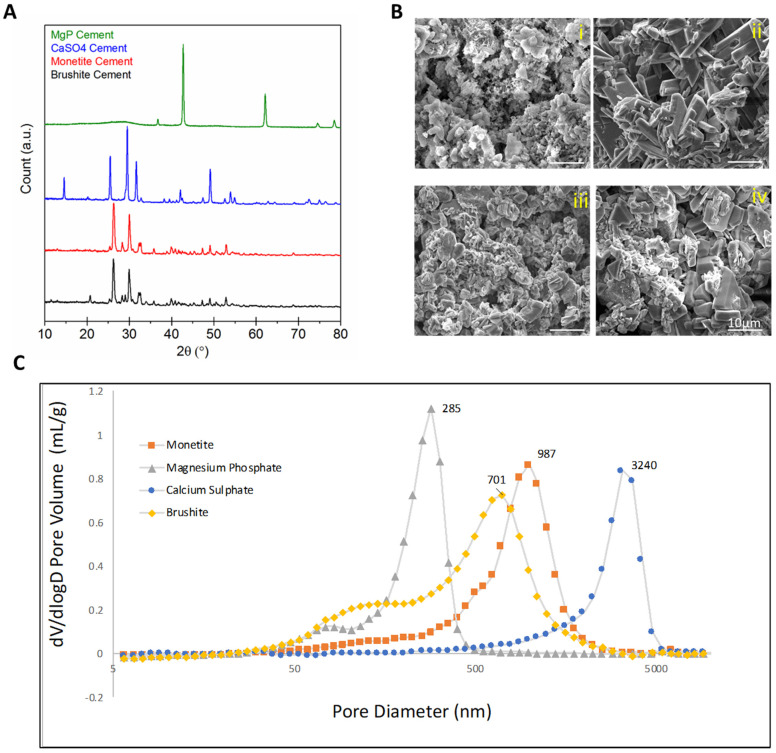
(**A**) X-ray diffraction patterns of the set cements after setting. (**B**) Scanning electron micrographs of fracture surfaces of (**i**) MgPO_4_, (**ii**) CaSO_4_, (**iii**) monetite, (**iv**) brushite. Calcium sulphate and brushite had similar microstructures whose cementitious matrix was made up of at least one population of platy crystals up to ~5 µm in dimension, whereas monetite and magnesium phosphate were more crystalline with rhomboid crystals >10 µm in dimension. (**C**) Mercury porosimetry showed the modal pore sizes lay in the range ¼ to 5 µm.

Since prior work had indicated that polymers could not elicit venous sprouting [14], here we examined the sprouting capacity of other inorganic materials that are in use clinically or have been proposed as bone graft substitutes. Analysis of the XRD patterns (Figure 7A) revealed an amorphous cementitious phase of magnesium phosphate, since only peaks characteristic of unreacted magnesium oxide at 43° and 62° 2θ were apparent. Indeed, all of the cements seemed to only partially react since only peaks of the starting material bassanite were visible in calcium sulphate cements, mostly tricalcium phosphate peaks were visible in monetite, and only a small peak at ~21° 2θ indicated that crystalline brushite was present in the brushite system. Representative SEM images of each group revealed similar porosity for all scaffolds (Figure 6B). In the same way, mercury porosity data (Figure 7C) revealed monomodal microporosities for all the materials in the range ¼ to 5 µm.

**Figure 8:**
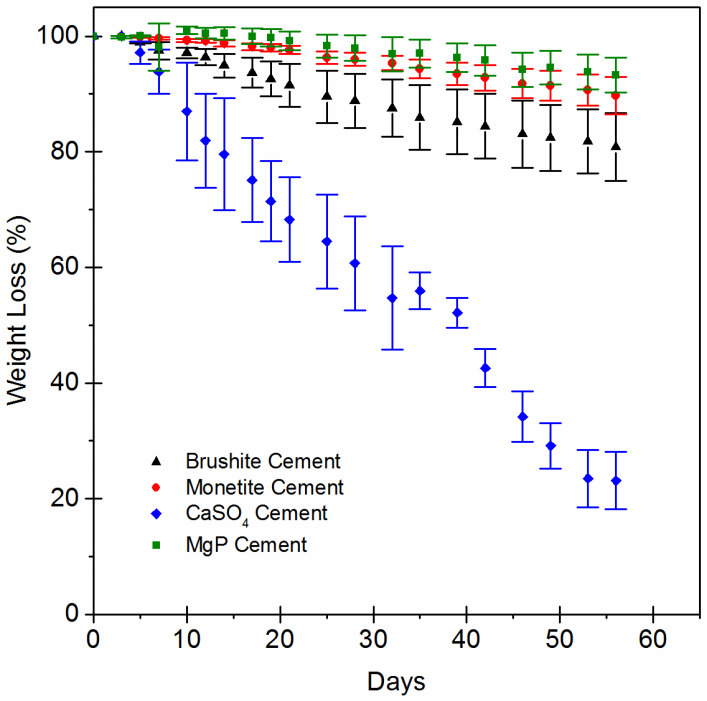
Results of weight loss of the four experimental materials used as scaffold in the present study: MgP, CaSO_4_, monetite, and brushite. The bars represent the standard deviation values for each measure (*n* = 5).

The weight-loss profiles of the scaffolds (Figure 8) showed similar degradation behaviour between monetite and brushite. Calcium sulphate, being the more soluble, had the greatest weight loss such that at 50 days in vitro, the sample had lost ~75% of its original weight. Brushite was the material with the second-highest weight loss, losing about 20% of its weight. Monetite lost only about 10% of its weight in this period, and magnesium phosphate lost about 7% of its weight over the course of the experiment.

**Figure 9:**
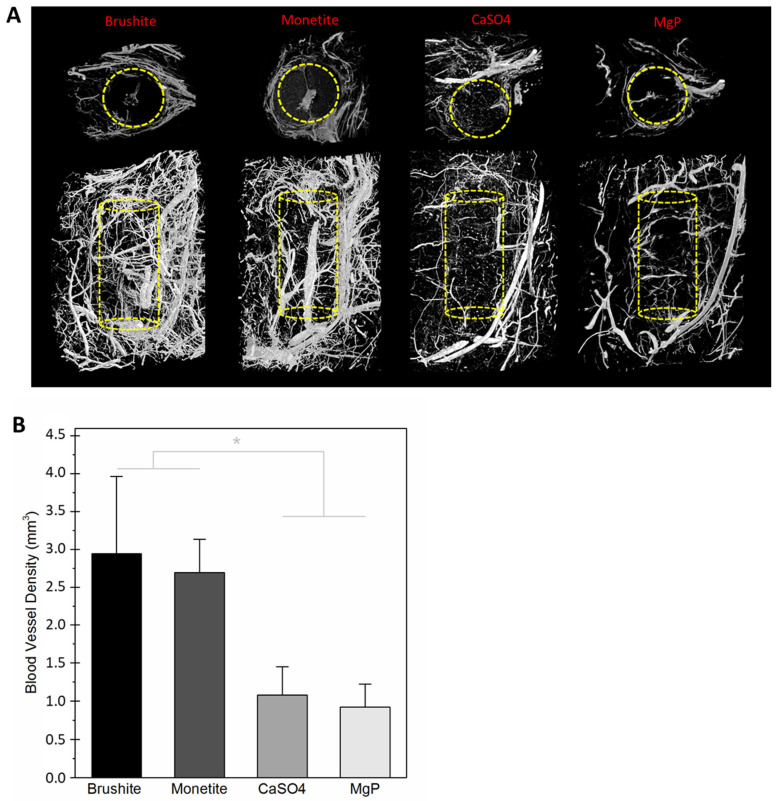
(**A**) Two-dimensional cross sections and 3D reconstructed images of vascular networks developed within and around scaffolds after 4 weeks of implantation, as shown by micro-CT. (**B**) Mean and standard deviation values of blood vessel density within scaffolds after 4 weeks of implantation (*n* = 4). There were neither statistically significant differences between brushite and monetite, nor between calcium sulphate and magnesium phosphate; however, these two groups were significantly different (* *p* < 0.05).

After harvest, the scaffolds were decalcified, allowing micro-CT imaging of the vasculature that had penetrated the scaffolds (Figure 9). In vitro dissolution was not predictive of in vivo dissolution, and all of the scaffolds were mostly intact with little discernible volume change. Quantification revealed that most vascular ingrowth was found in brushite (2.95 ± 1.2 mm^3^) and monetite (2.69 ± 0.5 mm^3^) scaffolds. Considerably fewer vessels were measured inside calcium sulphate (1.08 ± 0.43 mm^3^) or magnesium phosphate (0.93 ± 0.34 mm^3^) scaffolds.

Multiple attempts to perform IHC on these materials were hampered because magnesium phosphate and calcium sulphate microporosity was filled with an acellular matrix that was extremely fragile and disintegrated upon processing. Even within the fragments of matrix, no positive staining was observed; see Figure S4 for examples.

## 4. Discussion

In this study, we have begun to uncover stages in blood vessel response to microporous calcium phosphates. We found extensive infiltration of the cement by vSMC and possibly myofibroblasts (Figure 3 and Figure 4). This required both proliferation and migration into the scaffold. Myofibroblasts are known to accelerate capillary network formation via the formation of a supporting extracellular matrix [24].

vSMCs display various phenotypes and undergo phenotypic switching or plasticity. They produce the extracellular matrix that comprises the basement membrane and supports tensile strength and elasticity in vessels [25,26,27]. In response to endothelial-derived Notch signalling, vSMCs promote collagen synthesis [28], which is essential for maturation of vessels. The migration of vSMCs is primarily important in two physiological situations, i.e., recruitment of mural cells to the developing vessel and in response to vessel injury [29], and both seem relevant to this study. Endothelial Notch signalling has been shown to regulate expression of vSMC MMP-2 and MMP-9, which are important in the matrix remodelling necessary for cell migration through the vessel wall [30]. It is also important for recruitment of mural cells to the vessel wall in response to a PDGF gradient and maintaining their position via cellular adhesions.

The main chemoattractant for VSMCs is PDGF secreted by endothelial cells as well as platelets and macrophages [29,31]. The presence of macrophages mainly on the outside the scaffold after 2 weeks (Figure 5) could then have been one factor in driving migration of vSMCs. In addition, direct interactions between vSMCs and monocytes are known to stimulate expression of hepatocyte growth factor (HGF), a multifunctional factor implicated in tissue regeneration, wound healing, and angiogenesis [32]. Macrophage and vSMC crosstalk is also reported to induce angiogenesis [33,34]. Most of what is known about vSMCs, macrophages, and mineralised systems is in relation to pathological and often inflamed conditions. Here, vascularization of often calcified plaques is correlated with poor clinical outcomes [17,35,36,37]. However, interest is growing in potential angiogenic effects of inorganics, and recently, Wang et al. [16] found that a particular subset of macrophages were closely correlated to angiogenesis of calcium phosphate bioceramics and proved by selective depletion of CD301b+ macrophages that angiogenesis could be inhibited.

After the implantation of monetite, we observed that even after a week in vivo, there was an activation of NLRP3 and also of the cytokines Il-1β and Il-8 (Figure S3). NLRP3 activation [35] results in release of IL-1β, which in turn is known to upregulate IL-8 expression in vSMCs and vascular endothelial cells [38,39]. IL-8 itself is a known promotor of angiogenesis [40]. IL-1β is responsible for crosstalk between macrophages and smooth muscle cells and augments secretion of VEGF in both cell types [33]. In further support of the role of inflammatory processes, our laboratory previously observed that wrapping a silicone membrane around the outside of the implant prevented any luminal sprouting [14]. We tentatively attribute this to the prevention of phagocyte infiltration [41], since F4/80-positive cells were mainly observed in the outer portion of the scaffold initially (Figure 5).

Crystal recognition and subsequent activation of NLRP3 inflammasome is one mechanism behind what is known as biomaterials-driven sterile inflammation [42]. Better understanding the novel concept of biomaterials-driven sterile inflammation and associated damage-associated molecular patterns in long-term biodegradable implants is becoming recognized as important to improving tissue engineering and regenerative strategies. Bioactive ceramic chemical composition, porosity, and crystallinity affect biodegradation, inducing different host responses [43]. The cements tested as scaffolds in this study were chemically different and yet were all microcrystalline with broadly similar microporosities in the range 0.5–2 µm (Figure 8C).

Highly soluble calcium sulphate [44], a well-established bone graft substitute, did not result in cellular tissue ingrowth, just a very fragile matrix that did not withstand histological processing. The lack of in vivo dissolution may have resulted from fibrous encapsulation which would have limited ionic diffusion. It is speculated that the high calcium concentration in this material surrounded with a fibrous capsule may have prevented tissue ingrowth. Assuming that the fluid inside this scaffold was nearly saturated, the calcium levels would have approached ~25 mM [45], an order of magnitude higher than physiological levels. Since excessive calcium concentrations inhibit proliferation and migration and have been shown to slow healing [46,47,48], it is feasible that the microenvironment inside these materials was not conducive to cell migration or indeed survival [49]. Similarly, little vessel ingrowth was observed in magnesium phosphate. Relatively little is known about magnesium phosphates and their interactions with vSMCs, likely because these minerals rarely occur in the vascular system physiologically or pathologically. However, magnesium is a known inhibitor of NLRP3 activation, which may have contributed to the lack of vascularization observed [50,51].

Our temporal study in monetite showed a correlation between vasculature development and vSMC infiltration. Following injury caused by surgical placement, the calcium phosphate scaffold could support enhanced vSMC proliferation as the vessel wall healed. Macrophages and NLRP3 activation seem also to be implicated. There are many studies regarding the link between inflammation and vascular calcification in the vascular pathology literature. However, to our knowledge, this is the first study of blood vessel wall interactions with inorganic materials implanted directly. Given that to date there are no pro-angiogenic therapies in clinical use [52], the discovery that calcium phosphates can induce vascularization in vivo without microsurgery is intriguing. This study will assist in guiding optimization of inductive biomaterials. In the future, computational simulations could be used to improve the study of materials for tissue engineering, bringing advantages compared to laboratory studies. For example, in silico studies could make possible the accomplishment of several comparisons simultaneously, which cannot be performed with the same amount of time and financial supplies in vitro [53]. This approach would also allow for a more controlled and consistent environment, leading to more accurate and reproducible results.

The limitations of this study are that success in inducing neovascualture in rats does not necessarily imply that similar success could be expected clinically. While we did demonstrate that NLRP3 inflamasone was activated and that this was correlated with an angiogenic response, we did not show that the inflammasome activation caused angiogenesis. Future inhibition studies could confirm the mechanism. While there are reports vSMCs can phagocytose particles [35,54], we did not identify any evidence that this process was active. While we maintained porosity within a range, we did not take into account tortuosity. It is known that tortuosity also can have a profound effect on permeability [55] and could be expected to affect tissue ingrowth. While the induced blood vessels were functional for up to 6 weeks, a longer-term study would be required to confirm the stability of the neoagiosomes. There is intense interest by the vascular pathology research community in mechanisms by which calcified deposits can be resorbed in an attemt to develop non-invasive therapies, and future work will focus on this important phenomenon [56,57,58].

## 5. Conclusions

We show for the first time that microporous monetite scaffolds can support migration and proliferation of veinous smooth muscle cells. NLRP3 inflammasone activation was observed in cells within the scaffold with corresponding observation of IL-1β expression. It remains unclear whether blood vessels and the accompnaiying reparative cells in any way actively remodel the inorganic matrix or whether dissolution simply enlarges microporosity, allowing maturation of vessels. Further study can confirm whether NLRP3 activation is the single factor in initating luminal branching and neovascularization in microcrystalline calcium phosphates. Addtionally, the consideration of tortuosity and permeability in scaffolds of a single material type may yield additional valuable insights.

## Figures and Tables

**Table 1:** Powder to liquid (P:L) ratio used to produce each cement and the resultant porosity.

Scaffold	P:L Ratio (g/mL)	Porosity (%)
Brushite (CaHPO_4_·2H_2_O)	2:1	54.2 (±2.4)
Monetite (CaHPO_4_)	2:0.7	48.0 (±2.5)
Calcium sulphate (CaSO_4_·2H_2_O)	2:1	45.4 (±1.6)
Magnesium phosphate (MgHPO_4_)	1:1	50.6 (±7.0)

## Data Availability

The data presented in this study are available on request from the corresponding author.

## Supplementary Materials

The following supporting information can be downloaded at: https://www.mdpi.com/article/10.3390/jfb14020105/s1. Figure S1. Decalcified immunohistochemistry (IHC) showing CD34 staining around blood vessels in monetite scaffolds implanted for up to 6 weeks. The black substance inside vessels in 1 week and 6 week samples is Microfill contrast agent. Figure S2. Decalcified immunohistochemistry: The 1st and 4th weeks after implant with vein, the expression of α-SMA and Calponin-1. high magnification Scale bar 200 µm showing co-localisation of at least a population of the cells inside the scaffold. Figure S3. IHC of cells inside monetite scaffold at 1 wk. Rapid invasion by (A) F4/80+ (macrophages), (B) aSMA+, (C) Calponin-1+ (D) IL8+ cells, Scale bar 30 µm. Figure S4. Decalcified immunohistochemistry (IHC) staining of NLRP3 expression. (A) Brushite, (B) MgP, (C) CaSO_4_. Scale bars 1 mm.
