# Supplementary material for: Development of Neovasculature in Axially Vascularized Calcium Phosphate Cement Scaffolds
Source: J Funct Biomater. 2023 Feb 14;14(2):105. doi: 10.3390/jfb14020105 (PMC9966587; doi:10.3390/jfb14020105)
Supplement: Supplementary file 1 [file jfb-14-00105-s001.zip › jfb-2143090-supplementary.pdf]

SUPPLEMENTARY FIGURES

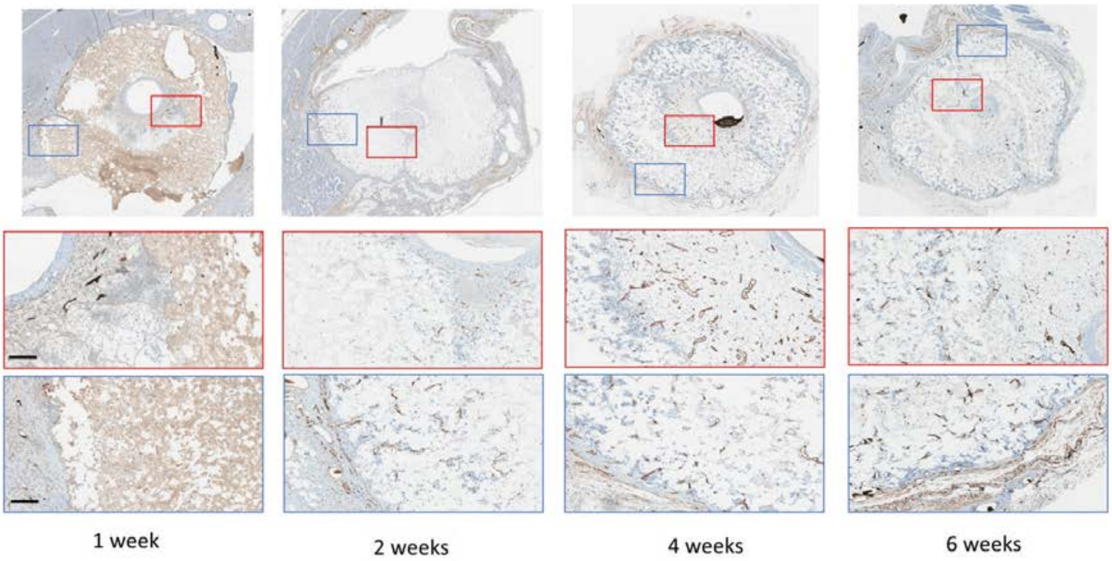

Figure S1. Decalcified immunohistochemistry (IHC) showing CD34 staining around blood vessels in monetite scaffolds implanted for up to 6 weeks. The black substance inside vessels in 1 week and 6 week samples is Microfill contrast agent.

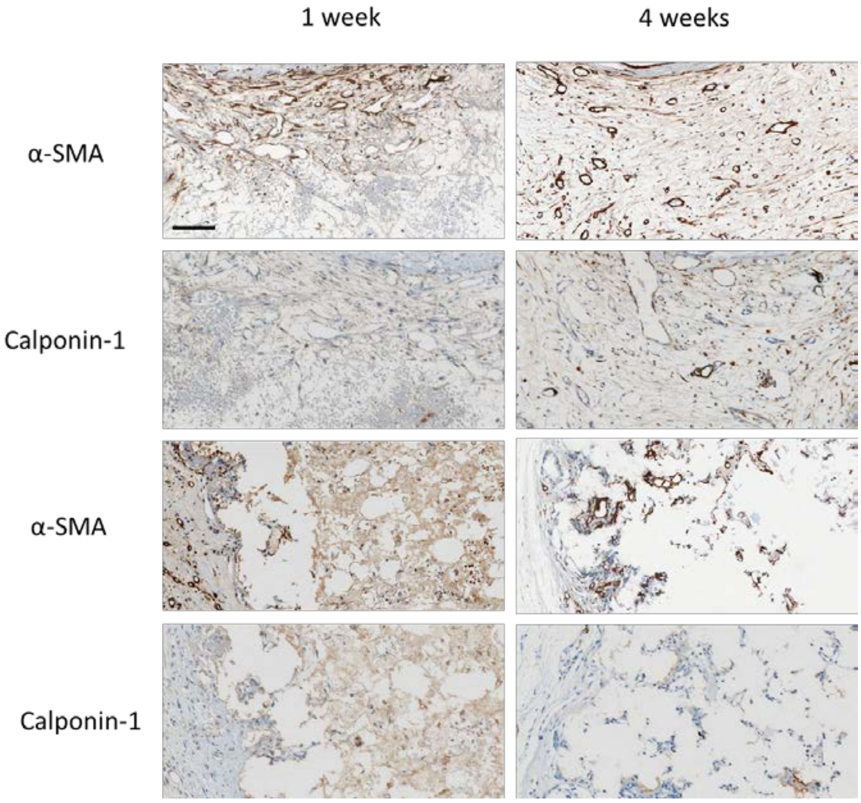

Figure S2. Decalcified immunohistochemistry: The 1st and 4th weeks after implant with vein, the expression of **α-SMA** and **Calponin-1.** high magnification Scale bar 200 µm showing co-localisation of at least a population of the cells inside the scaffold.

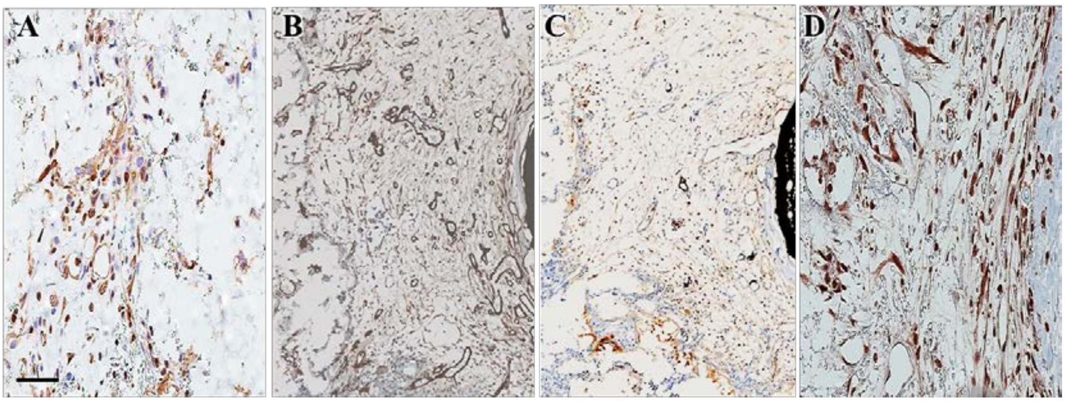

Figure S3. IHC of cells inside monetite scaffold at 1 wk. Rapid invasion by A) F4/80+ (macrophages,) B) aSMA+, C) Calponin-1+ D) IL8+ cells, Scale bar 30 µm.

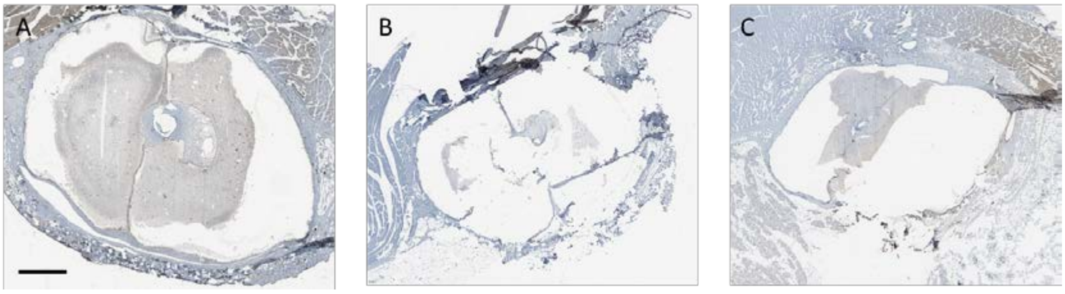

Figure S4. Decalcified immunohistochemistry (IHC) staining of NLRP3 expression. A) Brushite,B) MgP, C) $CaSO_4$. Scale bars 1 mm.
